# Real-time sampling of reasons for hedonic food consumption: further validation of the Palatable Eating Motives Scale

**DOI:** 10.3389/fpsyg.2015.00744

**Published:** 2015-06-01

**Authors:** Mary M. Boggiano, Lowell E. Wenger, Bulent Turan, Mindy M. Tatum, Maria D. Sylvester, Phillip R. Morgan, Kathryn E. Morse, Emilee E. Burgess

**Affiliations:** ^1^Department of Psychology, The University of Alabama at BirminghamBirmingham, AL, USA; ^2^Department of Physics, The University of Alabama at BirminghamBirmingham, AL, USA

**Keywords:** eating in absence of hunger, external factors, motivation, overeating, coping, emotional eating, obesity, stress

## Abstract

Highly palatable foods play a salient role in obesity and binge-eating, and if habitually eaten to deal with intrinsic and extrinsic factors unrelated to metabolic need, may compromise adaptive coping and interpersonal skills. This study used event sampling methodology (ESM) to examine whether individuals who report eating palatable foods primarily to cope, to enhance reward, to be social, or to conform, as measured by the Palatable Eating Motives Scale (PEMS), actually eat these foods primarily for the motive(s) they report on the PEMS. Secondly this study examined if the previously reported ability of the PEMS Coping motive to predict BMI would replicate if the real-time (ESM-reported) coping motive was used to predict BMI. A total of 1691 palatable eating events were collected from 169 college students over 4 days. Each event included the day, time, and types of tasty foods or drinks consumed followed by a survey that included an abbreviated version of the PEMS, hunger as an additional possible motive, and a question assessing general perceived stress during the eating event. Two-levels mixed modeling confirmed that ESM-reported motives correlated most strongly with their respective PEMS motives and that all were negatively associated with eating for hunger. While stress surrounding the eating event was strongly associated with the ESM-coping motive, its inclusion in the model as a predictor of this motive did not abolish the significant association between ESM and PEMS Coping scores. Regression models confirmed that scores on the ESM-coping motive predicted BMI. These findings provide ecological validity for the PEMS to identify true-to-life motives for consuming palatable foods. This further adds to the utility of the PEMS in individualizing, and hence improving, treatment strategies for obesity, binge-eating, dietary nutrition, coping, reward acquisition, and psychosocial skills.

## Introduction

While food is the body’s main source of fuel, eating for many individuals has become much more than a means of meeting one’s metabolic need. This is manifested by the many terms that have become commonplace in our vernacular and scientific literature, including “hedonic eating,” “emotional eating,” “stress-induced eating,” “food addiction,” “eating comfort foods,” “medicating with food,” “grazing,” etc. The types of foods that are typically eaten under these conditions are generally processed and made delicious by their high fat, sugar, and/or salt content. Correspondingly, their high palatability and caloric content compared to other foods promote overeating, a major cause of obesity and related metabolic disorders (Drewnowski, 1998; Yeomans et al., 2004; Lattemann, 2015). These types of foods are also craved, preferred, and difficult to limit so they tend to trigger and maintain binge-eating, a key feature of eating disorders such as bulimia nervosa and binge-eating disorder (White and Grilo, 2005; Bohon et al., 2009; Boggiano et al., 2013; Rigaud et al., 2014; Witt and Lowe, 2014). If eaten too frequently in place of meals, these foods can also undermine one’s nutritional health as they lack several essential nutrients, hence the term “junk-food” (Zizza et al., 2001; Poulos and Pasch, 2015). While links between these types of foods and obesity, metabolic syndromes, and eating disorders have been investigated, less attention has been paid to possible psychological consequences when individuals adopt the habit of eating as a way of dealing with unpleasant feelings or situations, of not seeking other sources than food for reward or comfort, and of failing to assert themselves in situations where social or conformity pressures preclude a healthier life-style for them. For example, we have noted that weight-loss patients commonly report that social expectations or reliance on food for reward or for coping reasons are significant barriers in maintaining their weight-loss. Yet, these factors have received little research attention. With this in mind, the Palatable Eating Motives Scale (PEMS) was developed to identify specific reasons for why individuals consume highly palatable foods and drinks (Burgess et al., 2014). It is a self-report questionnaire that yields four subscales or “motives”: Coping, Reward Enhancement, Social, and Conformity motives. However, like all psychometric instruments, the utility of the PEMS to prevent or treat these conditions is predicated on the ability of the PEMS to reflect an individual’s real-time experience, in this case, consuming palatable foods primarily for the motive identified by the PEMS.

Hence, the main goal of the present study was to test the validity of the PEMS by using event sampling methodology (ESM) of the event-contingent type to test if scores on the PEMS motives translate to real-life motives for eating palatable foods. That is, do individuals who score high on the Coping motive of the PEMS actually consume palatable foods or drink to deal with problems, worries, or a bad mood? Do those who score higher on the Reward Enhancement motive of the PEMS actually eat palatable food mainly for the pleasure, excitement, or increased fun of eating that food? ESM allowed us to determine if the frequency with which individuals report various motives for eating tasty foods is, in fact, reflected in the degree to which these motives actually drive palatable eating in their natural environment. Thus, ESM avoids the inaccuracies and biases that are related with global judgments measured by retrospective questionnaires. ESM also allows for within-person analyses that make it possible to examine factors occurring concurrently for any given participant. For example, it permitted us to take into account whether a participant’s motive for eating is higher or lower at times when that participant is overly stressed or eating during a weekend vs. weekday. Within-person associations can also have important theoretical and practical implications not otherwise revealed by between-person effects (Aﬄeck et al., 1999; Reis and Gable, 2000; Scollon et al., 2003).

**Table 1:** Participant characteristics and scores on the Palatable Eating Motives Scale (PEMS) motives and the event sampling methodology (ESM)-reported motives.

	Females		Males		Combined	
	*N*	Percent	*N*	Percent	*N*	Percent
	106	62.7	63	37.3	169	100
*Ethnicity*						
Whites	46	43.4	43	68.3	89	52.7
Blacks	41	38.7	10	15.9	51	30.2
Other	19	17.9	10	15.9	29	17.2

	**Mean**	**SD**	**Mean**	**SD**	**Mean**	**SD**

*Age*	21.07	4.14	21.16	3.70	21.10	3.97
*BMI*	27.50	7.24	27.71	6.52	27.60	6.98
*PEMS Subscales*						
PEMS Coping	**2.17**	**1.02**	**1.60**	**0.86^∗∗∗^**	1.96	1.00
PEMS Reward	2.15	0.90	2.08	0.89	2.01	0.89
PEMS Social	2.45	0.96	2.52	0.94	2.47	0.94
PEMS Conformity	1.56	0.62	1.55	0.61	1.56	0.60
*Number of eating episodes per participant*	**10.49**	**4.19**	**9.19**	**3.96^∗^**	10.01	4.14
*Event Sampling Subscales^a^*						
ESM-coping motive	**1.65**	**1.01**	**1.29**	**0.76^∗^**	1.51	0.94
ESM-reward motive	**2.10**	**1.49**	**1.65**	**0.97^∗^**	1.93	1.34
ESM-social motive	1.41	0.77	1.22	0.65	1.34	0.73
ESM-conformity motive	1.41	0.75	1.27	0.75	1.36	0.75
ESM-hunger motive	3.57	1.70	3.24	1.60	3.45	1.67
ESM-stress	**2.06**	**1.29**	**1.66**	**0.96^∗^**	1.91	1.19

^∗^*p* < 0.05, ^∗∗∗^*p* < 0.001 females vs. males; SD.

^a^Mean score for each ESM subscale computed from the average ESM score for each participant weighted by the number of eating events reported by the participant (*n* = 1691 palatable eating events). Bolded values denote significant sex differences.

Data were collected from college students during actual eating events over a 4-day period that included a weekend. We expected that scores on each PEMS motive would be significantly and most strongly correlated with scores on the corresponding ESM-reported PEMS motive. To increase the degree of validity of the PEMS that would be provided by associations with real-time motives, the ESM version of the PEMS included physical hunger as an additional motive for eating the tasty foods/drinks. Since college students are likely to choose highly palatable foods when hungry as well as when not hungry, hunger could be endorsed as a motive for eating palatable foods. However, we expected scores on the ESM-reported motives and the PEMS motives to be strongly correlated despite the choice to report hunger as a motive, even as a primary motive on some days or eating events. The validation test for the PEMS instrument via ESM was additionally strengthened by having students record and submit the types of tasty food and/or drinks consumed during each eating event. This would allow verification that the foods being eaten adhered to the PEMS defined “tasty foods and drinks.” Moreover, recorded foods and drinks allowed us to confirm that instructions were followed and that the eating event did indeed include palatable food and drink items. Lastly, because stress is known to affect food intake (Bazhan and Zelena, 2013), the ESM version of the PEMS also included an item assessing the subjective level of stress experienced at the time of the eating event.

A secondary aim in this study was to test if the link between the PEMS Coping motive and body mass index (BMI) would be replicated in the present sample of college students and, more importantly, if it would replicate when using the real-time (ESM-reported) coping motive^1^. Previous studies in college students and weight-loss seeking patients have consistently found that higher frequency to eat for Coping motives as assessed with the PEMS is associated with greater BMI independent of binge-eating status as measured by the Binge-Eating Scale, food addiction scores as measured by the Yale Food Addiction Scale, scores on the other PEMS motives, and demographics such as age, sex, and ethnicity (Boggiano et al., 2014; Burgess et al., 2014). Also changes in Coping scores over a 2-year period in college students predicted a change in BMI where a 1-point increase or decrease in Coping scores predicted a 10.5 lb. gain or loss, respectively (Boggiano et al., 2015). Thus, in the present study we expected that eating for Coping motives during actual eating events would also be a significant predictor of BMI. Overall, the results of the present study should strengthen the validity of the PEMS as an instrument that can predict real-world psychological motives for eating foods linked to obesity, binge-eating, poor nutrition, and as substitutes for otherwise healthier coping, reward acquisition, and inter-social skills.

## Materials and Methods

### Participants

Undergraduate students were recruited from Introduction to Psychology course participant pools at The University of Alabama at Birmingham (UAB) and were offered research credit for their participation. Psychology students from other courses were recruited through flyers and were compensated with a $60 Visa gift card. Exclusionary criteria included pregnancy, an age younger than 19, and a measured BMI of less than 18 kg/m^2^. The 169 students participating in the present protocol were also part of a larger study. This undergraduate student sample included males and females of diverse ethnicities with an average age of 21.1 years (SD = 3.97, 91.7% between ages of 19 and 25). A more detailed description of the participants is provided in **Table 1**. This study was approved by the UAB Institutional Review Board for Human Use.

### Measures

#### Body Mass Index

After removal of shoes, weight and height for each non-fasted participant were measured by a research assistant in a private room. BMI was calculated with the formula: weight in kg/(height in meters)^2^.

#### Palatable Eating Motives Scale

The PEMS is a 19-item^2^ self-report questionnaire that assesses how frequently one consumed tasty foods and drinks in the past year for various reasons. Each item uses a 5-point Likert-like scale from 1 = “Almost never/never” to 3 = “Half the time” to 5 = “Almost always/Always.” Examples of tasty foods/drinks are provided in the instructions under the general categories of fast foods, homemade fried foods, sweets, salty snacks, and non-alcoholic sugary drinks (see Burgess et al., 2014 for the original survey^2^). These PEMS items factor into one of four motives: Coping (e.g., “To forget about your problems”), Reward Enhancement (e.g., “Because it gives you a pleasant feeling”), Social (e.g., “To celebrate a special occasion with friends”), and Conformity (e.g., “Because your friends or family want you to eat or drink these foods or drinks”). These motives have good internal consistency and test–retest reliability (Burgess et al., 2014; Boggiano et al., 2015). Motive scores are calculated by computing the mean of responses across items that comprise each motive.

#### Eating Events, Food Diary, and Survey

Participants were given a paper diary with sufficient space to record multiple eating events for four consecutive days. Each sheet repeated the examples of “tasty foods and drinks” that were in the PEMS instructions to remind the participant of what was meant by “tasty foods and drinks.” They were instructed to keep the diary with them at all times and record the day and time of day when they consumed tasty foods and/or drinks regardless of whether it was a meal, snack, or dessert. They were instructed to write down what tasty foods/drinks they consumed and to answer a brief six-item survey as to “why or how you felt when you consumed the foods and drinks at this time.” Participants were requested to do this during or as soon after the eating event as possible. The first four questions were the ESM version of the four PEMS motives. Each question combined all of the items that comprised the PEMS motive into one question. For example, the ESM coping question read, “To help me forget about my worries; or to feel less depressed, nervous, or stressed; or to cheer me up from a bad mood; or to forget about my problems.” The fifth question treated hunger as an additional possible motive and read, “Because I was physically (stomach) hungry; or to take away hunger pains; or because I knew it would be a long time before my next meal and wanted to prevent getting hungry.” These five questions were followed by a 5-point response scale from 1 = “Not at all the reason” to 3 = “Somewhat the reason” to 5 = “Definitely the reason.” The last question was not a motive query but asked “How stressed did you feel around the time that you ate the above?” and used a 5-point response scale from 1 = “Not at all the stressed” to 3 = “Somewhat stressed” to 5 = “Extremely stressed.” This set of six questions was completed by participants every time they consumed a tasty food(s) and/or drink(s). Participants could enter as many eating events as occurred during a day for the 4 days.

### Procedures

The study involved two visits to the laboratory. In the first visit, participants completed the consent process, were measured for a BMI, provided demographic data, and completed an electronic version of the PEMS in a private room. Participants were then instructed on how to complete a paper version of the food diary including the set of six questions for each palatable eating and/or drinking event. Participants were instructed to start recording their eating event information for four consecutive days starting on Thursday and ending on Sunday. No participants were seen during holidays and they were instructed not to complete the food diaries during any consecutive 4-day period that included a holiday, e.g., Thanksgiving or Christmas, because their normal eating habits were likely to be altered. At the end of each day, the participants were asked to log on to the study website and transfer the foods/drinks eaten at each event and the corresponding responses to the six questions from the paper diary to an electronic survey that mirrored the paper version. They were also tutored on a demo of this electronic survey during their first visit in the lab so that they would be familiar with it when they logged on from their home computers to enter the information. If they consumed no tasty foods or drinks on a given day, they had the option of clicking “none” on the electronic survey. Once electronic responses for all 4 days were received, the students were scheduled for a second visit to turn in their paper food diaries and to complete additional questionnaires for a different study.

### Statistical Analyses

Since we previously found sex differences in PEMS motive scores in a large (*N* = 1478) sample of college students (unpublished data), separate MANOVAs assessed differences between females and males on the PEMS motive scores and the ESM-reported variables. An ANOVA assessed sex differences in the number of eating episodes. Since the number of eating events varied across participants, means for the ESM-motives and other ESM-variables were computed from their corresponding average ESM score for each participant weighted by the number of eating events reported by the participant (the multiplicative weighting factor = number of reported events for a participant/total number of reported events from all participants). Two-level linear mixed models (eating events nested within persons) were used to estimate the likelihood that each eating event-reported motive would be significantly associated with its corresponding PEMS motive. Predictors of the ESM-motives at level 1 (within-person level) included ESM-reported hunger and stress scores for each eating event along with whether the eating event occurred on a weekend day (Thursday or Friday = 0, Saturday or Sunday = 1). Level 2 (between-person level) predictors were person-specific variables and included PEMS motive scores, age, sex, ethnicity, and BMI. These person-specific predictors, except for the dichotomous sex (female = 0, male = 1) and ethnicity variables, were centered using their grand mean (difference between a participant’s score or value and the mean score or value across all participants for a specific variable) prior to analysis. As an example of the mixed modeling equations, the level-1 equation for ESM predictors of the ESM-coping motive was:

(1) $$\begin{array}{l} \mathrm{ESM-coping}=\beta_{0}+\beta_{1}{}^{*}(\mathrm{Weekend})\\ {\mathrm{+\beta}}_{2}{}^{*}(\mathrm{ESM-hunger})\\ \;\;{\mathrm{+\beta}}_{3}{}^{*}(\mathrm{ESM-stress})\mathrm{+ r} \end{array}$$

The corresponding level-2 equations for person-specific predictors of level-1 parameters (β) were:

(2) $$\begin{array}{l} \beta_{0}=\gamma_{00}+\gamma_{01}{}^{*}(\mathrm{Age})\;+\gamma_{02}{}^{*}(\mathrm{Sex})\;{\mathrm{+ \gamma}}_{03}{}^{*}(\mathrm{Ethnicity}1)\\ {\mathrm{+\gamma}}_{04}{}^{*}(\mathrm{Ethnicity}2)\;+\gamma_{05}{}^{*}(\mathrm{BMI})\\ {\mathrm{+\gamma}}_{06}{}^{*}(\mathrm{PEMS Coping})\;+\gamma_{07}{}^{*}(\mathrm{PEMS Reward})\\ {\mathrm{+\gamma}}_{08}{}^{*}(\mathrm{PEMS Social})\;+\gamma_{09}{}^{*}(\mathrm{PEMS Conformity})\mathrm{+ \mu}\\ \beta_{1}=\gamma_{10}\\ \beta_{2}=\gamma_{20}\\ \beta_{3}=\gamma_{30} \end{array}$$

Finally, separate linear regression models were used to determine if PEMS motive scores and if ESM-motive average scores predicted BMI as the dependent variable while controlling for demographics. To account for the different number of eating events reported by each participant, the linear regression for the ESM-motives also controlled for the number of eating events and for the interactions between each average ESM-motive score and the number of eating events. All non-dichotomous variables in the regression models were centered using their grand mean prior to analysis. All analyses including the mixed modeling were conducted via SPSS version 22.

## Results

Prior to any calculations and analyses involving the ESM-reported variables of coping, reward enhancement, social, conformity, hunger, and stress from the six questions of the food diary, the recorded foods and drinks for each eating event were checked to ensure that the eating event actually included palatable food and drink items as described in the PEMS and paper food diary instructions. Out of a total of 1734 reported eating events, *n* = 1691 (97.5%) included palatable items and were included in the data analyses.

### Participant Characteristics

**Table 1** provides sex, ethnicity, and age descriptions of the sample. The mean BMI of the participants was in the overweight range, 27.6 kg/m^2^, and did not differ between males (27.7, SD = 6.5) and females (27.5, SD = 7.2). Likewise mean age did not differ between sexes. **Table 1** also lists the mean scores for males and females on the PEMS motives and the ESM motives in addition to the hunger and stress items. While females generally scored higher than males on both the PEMS and ESM-reported motives, only the Coping motive scores (both ESM-reported and PEMS) and ESM-reported reward enhancement scores were significantly higher in females. Also notable is that while participants ate more frequently for Social motives than any other PEMS motive, the ESM-social motive was, numerically, the least of the ESM motives endorsed. Lastly, the average number of eating events per person over the 4-day period was 10.01 (SD = 4.14, range: 2–23) when both sexes were combined. As also noted in **Table 1**, females reported significantly more eating events and feeling more stressed than males on the ESM reports.

### Associations between the PEMS Motives and the ESM-Reported Motives

As shown by boldface type in **Table 2**, the highest correlation within the PEMS motives was with their respective ESM motives. Specifically, frequency to eat for Coping motives on the PEMS (PEMS Coping, γ_06_) was significantly associated with the real-time or ESM-reported reasons to eat for coping motives, independent of ESM-hunger as a motive and perceived levels of stress during the eating event. For every 1-point increase in the PEMS Coping motivation frequency, there was a 0.20 increase on the 1–5 scale of the ESM reported reason for eating to cope. Also as predicted, the PEMS Reward Enhancement and PEMS conformity motives predicted the ESM-reward enhancement and conformity motives, respectively. For every 1-point increase in these motivations, there was a respective 0.46 and 0.28 increase in their corresponding ESM-motives. Lastly, the PEMS Social motive was modestly associated (*b* = 0.13, *p* < 0.017) with the ESM reporting of eating tasty foods for social reasons.

**Table 2:** Results from two-level mixed models predicting ESM-reported motives from the PEMS and covariates.

				Dependent measure
	
	ESM-coping			ESM-reward			ESM-social			ESM-conformity		
Variables	*b*	SE	*p*	*b*	SE	*p*	*b*	SE	*p*	*b*	SE	*p*
**For intercept, β_**0**_**												
Intercept, γ_00_	1.063	0.080	-	2.683	0.131	-	1.801	0.092	-	1.679	0.094	-
Age, γ_01_	0.009	0.008	0.297	0.045	0.016	0.005	0.002	0.009	0.860	-0.004	0.009	0.648
Sex, γ_02_	0.051	0.072	0.476	-0.035	0.138	0.802	-0.019	0.079	0.808	0.028	0.080	0.726
Ethnicity 1, γ_03_	0.015	0.077	0.847	-0.036	0.148	0.806	-0.086	0.085	0.310	-0.059	0.086	0.491
Ethnicity2, γ_04_	0.034	0.091	0.714	-0.151	0.174	0.387	-0.169	0.101	0.096	0.005	0.102	0.958
BMI, γ_05_	-0.003	0.005	0.542	-0.015	0.009	0.122	-0.001	0.005	0.813	0.002	0.005	0.680
PEMS Coping, γ_06_	**0.202**	**0.039**	**<0.001**	0.018	0.075	0.814	0.029	0.043	0.502	0.022	0.044	0.620
PEMS Reward, γ_07_	0.056	0.044	0.209	**0.455**	**0.084**	**<0.001**	0.021	0.049	0.672	0.019	0.049	0.704
PEMS Social, γ_08_	0.013	0.047	0.788	-0.093	0.090	0.307	**0.126**	**0.052**	**0.017**	0.087	0.053	0.102
PEMS Conformity, γ_09_	0.078	0.073	0.290	0.009	0.090	0.948	0.032	0.081	0.693	**0.284**	**0.082**	**<0.001**
**For Weekend slope,**β_1_												
Intercept, γ_10_	0.020	0.031	0.521	0.090	0.040	0.025	0.095	0.038	0.013	0.120	0.039	0.002
**For ESM-hunger slope,** β_2_											
Intercept, γ_20_	-0.105	0.011	<0.001	-0.228	0.015	<0.001	-0.079	0.014	<0.001	-0.070	0.014	<0.001
**For ESM-stress slope,** β_3_											
Intercept, γ_30_	0.401	0.018	<0.001	0.007	0.025	0.777	-0.091	0.022	<0.001	-0.066	0.022	0.003

Coefficients of fixed effects in the table are unstandardized. Dashes indicate no *p* value for the intercept because the model evaluates the difference from zero but the response scale does not contain a zero point (responses are coded 1–5, not 0–4) Sex: dummy-coded male = 1/female = 0; Ethnicity1: dummy-coded Blacks = 1/Whites = 0; Ethnicity2: Others = 1/Whites = 0; Weekend: dummy-coded Saturday or Sunday = 1/Thursday or Friday = 0. Bolded values denote significant associations between PEMS and respective ESM-reported motives.

Of note, the ESM-hunger motive for eating tasty foods/drinks was significantly associated (*p* < 0.001) with all four ESM-motives but in a negative direction such that lower scores on eating for hunger corresponded to higher real-time scores on eating for coping, reward enhancement, social, and conformity motives. On the other hand, ESM-reported stress level had a significant, positive association with the ESM-coping motive; no significant association with ESM-reward enhancement; and a significant negative association with the ESM-social and conformity motives. Finally, consuming tasty foods and/or drinks on the weekend had a significant positive association with the ESM-reward enhancement, social and conformity motives, but no association with the ESM-coping motive for consuming tasty foods or drinks.

### Association between PEMS Coping and ESM-reported Coping on BMI

A secondary aim in this study was to test if the association between the Coping motive and BMI would be replicated in the present sample of college students, and if it would replicate using real-time coping motive scores. As shown in **Table 3**, older age not surprisingly predicted increased BMI. More importantly, PEMS Coping was the only motive of the four motives to account for variance in BMI and, in a separate regression model, ESM-coping was also found to significantly account for variance in BMI. Both PEMS Coping and ESM-coping scores predicted BMI at a similar level (β = 0.21) and significance (*p* < 0.05).

## Discussion

The primary goal of this study was to test the validity of the PEMS by determining if the frequency with which individuals report various motives for eating tasty foods or drinks is reflected in the motives they experience when actually eating these foods and drinks. As predicted, each of the PEMS motives was only correlated with its corresponding real-time ESM-motive, even when eating for hunger and perceived stress levels were included in the ESM analyses. That is, while several participants endorsed hunger as the main motive in certain eating events and while they reported higher and lower levels of stress during some eating events, these factors did not abolish the significant association between the PEMS and its corresponding ESM motive. Therefore, scores on the PEMS motives should confidently reflect reasons for eating palatable foods in the real world regardless of varying states of hunger or stress.

**Table 3:** Linear regression models of the mean ESM-reported motive scores and PEMS scores with BMI as the dependent variable.

Dependent variable	BMI					
	ESM-motive scores^a^			PEMS motive scores		
Independent variables	*β*	*t*	*p*	*β*	*t*	*p*

**Demographics**						
Age	**0.189**	**2.37**	**0.019^∗^**	**0.161**	**2.16**	**0.032^∗^**
Sex	0.068	0.84	0.402	0.100	1.26	0.208
Ethnicity 1	0.117	1.39	0.166	0.124	1.54	0.126
Ethnicity2	-0.020	-0.24	0.810	-0.013	-0.16	0.871
**Motive subscales**						
Coping	**0.202**	**2.41**	**0.017^∗^**	**0.207**	**2.35**	**0.020^∗^**
Reward	-0.051	-0.62	0.535	0.106	1.19	0.237
Social	-0.009	-0.10	0.920	0.071	0.70	0.485
Conformity	0.041	0.42	0.673	0.013	0.13	0.895

Regression coefficients are standardized and all variables were centered using their grand mean prior to analysis. Sex: dummy-coded male = 1/female = 0; Ethnicity1: dummy-coded Blacks = 1/Whites = 0; Ethnicity2: Others = 1/Whites = 0.

^a^Mean scores for ESM-reported motives computed from group means for each participant. Analyses controlled for number of eating episodes and the interaction between the number of eating episodes and ESM-reported mean scores.

^∗^Significant predictors, *p* < 0.05 and bolded.

Stress was found to be positively associated only with the ESM-coping motive suggesting that individuals who habitually eat tasty foods to cope may be more susceptible to use these foods to deal with stress. Conversely, stress may also be more likely to trigger increased intake of tasty foods in those who primarily eat to cope. This is supported by others who found that “emotional eaters” or individuals that eat to escape negative situations tend to eat more when under stress (Oliver et al., 2000; Wallis and Hetherington, 2004). Also the PEMS Coping motive has previously been found to correlate highly with perceived stress reactivity and urges to eat triggered by negative emotions (unpublished data). Therefore, the link between stress and coping found in the ESM results adds construct validity to the Coping motive. A limitation of the present study is that the degree of dietary restriction was not assessed as it is a common mediator of stress-induced eating (Oliver et al., 2000; Wallis and Hetherington, 2004; Boggiano et al., 2005) and possibly a mediator of eating to cope. However, dietary restriction may not be necessary for eating to cope given that the Restraint scale scores from the Eating Disorders Examination Questionnaire in a separate study were found to be uncorrelated with Coping scores (unpublished data) and may be a reason why higher Coping scores predict higher BMI (Boggiano et al., 2015).

Interestingly, reported stress during eating events was also a significant predictor of ESM-reported social and conformity motives but it was a negative predictor. Indeed, stress-induced undereating is a normal and physiologically adaptive response in the short-term (Chrousos, 1998; Bazhan and Zelena, 2013). Coupled with previous evidence for Social and Conformity motives to be the least associated with obesity and binge-eating (Boggiano et al., 2014, 2015; Burgess et al., 2014), this negative association with stress supports the normative nature of these motives compared to the Coping and Reward Enhancement motives.

The inclusion of hunger as an additional motive in the ESM survey also yielded interesting results. We expected that eating tasty foods for hunger would not be as frequently endorsed as the other motives but the inclusion of fast foods and fried foods in the instructions, especially in a college sample, and the fact that we did not exclusively sample for snack vs. meals may have resulted in the high endorsement of hunger as a motive. Indeed, hunger was the most frequent reason for eating tasty foods as evidenced by a mean of score of 3.45 for the ESM-hunger motive shown in **Table 1**. Nevertheless, scores on the ESM-reported motives and the PEMS motives were still strongly correlated in spite of the inclusion of hunger as a motive, providing further validity for the PEMS. Furthermore, this validation is strengthened by the fact that hunger was a significant negative predictor of all four ESM-reported motives. That is, the lower the reason that hunger was attributed as the reason for eating tasty foods, the greater the reason that one of the PEMS motives was behind the eating.

Another important result of the study was that the weekend days of Saturday and Sunday significantly predicted the ESM-reward enhancement, social, and conformity motives. This adds construct validity for the PEMS and confirms the veracity of participant responding given that social and conformity motives are influenced by social gatherings and interactions with others which should be more prevalent on the weekend than during the week days for college students. The students might also be expected to eat more foods for pleasure (reward enhancement) when out of their daily week-day routine of classes and work. In stark contrast, weekend days did not affect the ESM-coping motive, suggesting that eating to cope may be a more trait- vs. state-related motive. Some support for this is that the Coping has the highest test–retest reliability of the PEMS motives (Boggiano et al., 2015).

The secondary hypothesis of this study was also confirmed in that the positive association between the PEMS Coping scores and BMI previously reported in student and weight-loss seeking populations (Boggiano et al., 2014, 2015; Burgess et al., 2014) was replicated in this sample of college students. More importantly, it was replicated using the real-time coping scores. In addition, the coefficients from the present regression models were very similar to previously reported values on different college student samples (Burgess et al., 2014; Boggiano et al., 2015). Thus the ESM-coping motive did not provide additional information beyond what the PEMS Coping motive did in predicting BMI. Consequently, scores on the PEMS alone are sufficient to predict BMI.

While ESM validated the generalization of PEMS motive scores to real-life reasons for eating tasty foods, the ESM scores for the motives were lower than their respective PEMS scores. This can be seen in **Table 1**. One reason for this may be that the ESM survey included perceived hunger as an alternate motive. Without this motive, participants would be restricted only to the four motives represented in the PEMS which might have led to higher means for the ESM motives. Likewise, the inclusion of the perceived stress level in the ESM survey and its positive association with the ESM-coping motive would have and did reduce the fixed effect coefficient (magnitude of γ_06_ in **Table 2**) between the PEMS Coping and ESM-coping motive scores. Another reason for the lower mean motive scores and fixed effect coefficients γ_06-_γ_09_ might have been due to the fact that the PEMS measures the frequency with which individuals eat tasty foods (“almost never/never” to “almost always/always”) for a particular motive while the ESM version of the PEMS measured the degree to which a certain motive was the reason for eating the tasty foods at the time they were eaten (“not at all the reason” to “definitely the reason”). Also the ESM reflected a summary of eating events over a 4-day timeframe whereas the PEMS questionnaire inquires about motives over the past 12 months. The PEMS also used 4–5 items to assess each motive while the ESM used only one collective item to assess each motive. This was done to avoid response bias and fatigue, especially if numerous events were reported per day, but it may have contributed to lower associations between the ESM and PEMS scores. The impact of these differences on lower ESM-motive scores and fixed effect coefficients could have been decreased by obtaining a greater number of eating events per person. As the number of eating events increases, the mean of an ESM motive score for an individual should become more reflective of the individual PEMS-frequency based score. This could be remedied by allowing the event reporting to extend to more than 4 days. This 4-day limitation may be especially important in explaining the much lower ESM-social mean than the PEMS Social mean in **Table 1**, and the lower beta between the PEMS Social and ESM-social motives vs. beta values for the other motives in **Table 2**. We suspect this is due to the varying nature of social eating itself. Of all the motives Social is the most susceptible to situational factors such as birthdays, special occasions, or celebrations other than major holidays which we were not able to control. Participants may have had fewer social gatherings or social settings to eat tasty foods and drinks during the 4-day window when data were collected than in the 12-month period they are asked to keep in mind when completing the PEMS.

In conclusion, the results of this ESM study provide ecological validity for the PEMS as an instrument that can be used to identify an individual’s real-life motive(s) behind consuming palatable foods and drinks. The results also evidence the robustness of the PEMS as a measure of real-life motives for consuming palatable foods because the ESM and PEMS motives correlated despite varying levels of hunger and stress surrounding eating events. For the Coping motive, previously reported associations with BMI were strengthened in this study not only by replication but by an association between real-time coping motives and BMI. The results also confirm that the PEMS measures eating in the absence of hunger because all four motives during real-time assessment were significantly correlated with decreased eating for hunger. This adds to the potential consequences of eating for PEMS-identified motives. Hence identifying and targeting one’s primary PEMS motive should improve treatments for obesity and binge-eating and improve nutrition. At the same time, knowledge of one’s primary motive or motives can be used to help individuals adopt healthier ways of coping, obtaining reward, and interacting with others than through intake of food.

## Conflict of Interest Statement

The authors declare that the research was conducted in the absence of any commercial or financial relationships that could be construed as a potential conflict of interest.

## Author Contributions

The study was conceived by MB and EB; data was acquired by MT, MS, KM, and PM and analyzed by MB, LW, and BT. MB and LW drafted the manuscript and critical reviews were provided by all other authors. All authors approved the final version and agreed to be accountable for all aspects of the work.

## Footnotes

1 ESM-reported motives will be spelled out with small-case letters, e.g., “coping” while PEMS motives will be capitalized, e.g., “Coping.”

2 A revised 20-item version of the PEMS is now in use. It includes an additional item that factored with the Coping motive so that now all four motives are comprised of five items. A copy of this version can be requested from the corresponding author.

## References

[B1] Aﬄeck G. Zautra A. Tennen H. Armeli S. (1999). Multilevel daily process designs for consulting and clinical psychology: a preface for the perplexed. *J. Consult. Clin. Psychol*. 67 746–754. 10.1037/0022-006X.67.5.746 10535241

[B2] Bazhan N. Zelena D. (2013). Food-intake regulation during stress by the hypothalamo-pituitary-adrenal axis. *Brain Res. Bull.* 95 46–53. 10.1016/j.brainresbull.2013.04.002 23590931

[B3] Boggiano M. M. Burgess E. E. Turan B. Soleymani T. Daniel S. Vinson L. D. (2014). Motives for eating tasty foods associated with binge-eating: results from a student and a weight-loss seeking population. *Appetite* 83 160–166. 10.1016/j.appet.2014.08.026 25169880 PMC4962333

[B4] Boggiano M. M. Chandler P. C. Viana J. B. Oswald K. D. Maldonado C. R. Wauford P. K. (2005). Combined dieting and stress evoke exaggerated responses to opioids in binge-eating rats. *Behav. Neurosci.* 119 1207–1214. 10.1037/0735-7044.119.5.1207 16300427

[B5] Boggiano M. M. Turan B. Maldonado C. R. Oswald K. D. Shuman E. S. (2013). Secretive food concocting in binge eating: test of a famine hypothesis. *Int. J. Eat. Disord.* 46 212–225. 10.1002/eat.22077 23255044 PMC5098405

[B6] Boggiano M. M. Wenger L. E. Turan B. Tatum A. A. Morgan P. R. Sylvester M. D. (2015). Eating tasty food to cope: longitudinal association with BMI. *Appetite* 87 365–370. 10.1016/j.appet.2015.01.008 25596500 PMC4951003

[B7] Bohon C. Stice E. Burton E. (2009). Maintenance factors for persistence of bulimic pathology: a prospective natural history study. *Int. J. Eat. Disord.* 42 173–178. 10.1002/eat.20600 18951457 PMC2642526

[B8] Burgess E. E. Turan B. Lokken K. L. Morse A. Boggiano M. M. (2014). Profiling motives behind hedonic eating. Preliminary validation of the Palatable Eating Motives Scale. *Appetite* 72 66–72. 10.1016/j.appet.2013.09.016 24076018

[B9] Chrousos G. P. (1998). Stressors, stress, and neuroendocrine integration of the adaptive response. The 1997 Hans Selye Memorial Lecture. *Ann. N. Y. Acad. Sci.* 851 311–335. 10.1111/j.1749-6632.1998.tb09006.x 9668623

[B10] Drewnowski A. (1998). Energy density, palatability, and satiety: implications for weight control. *Nutr. Rev.* 56 347–353. 10.1111/j.1753-4887.1998.tb01677.x 9884582

[B11] Lattemann D. F. (2015). Modulation of food reward by endocrine and environmental factors: update and perspective. *Psychosom. Med*. 10.1097/PSY.0000000000000146 [Epub ahead of print]. PMC4501879 25738439

[B12] Oliver G. Wardle J. Gibson E. L. (2000). Stress and food choice: a laboratory study. *Psychosom. Med*. 62 853–865. 10.1097/00006842-200011000-00016 11139006

[B13] Poulos N. S. Pasch K. E. (2015). Energy drink consumption is associated with unhealthy dietary behaviours among college youth. *Persp. Public Health* 10.1177/1757913914565388 [Epub ahead of print]. 25667166

[B14] Reis H. T. Gable S. L. (2000). *Handbook of Research Methods in Social and Personality Psychology*. New York: Cambridge University Press.

[B15] Rigaud D. Jiang T. Pennacchio H. Brémont M. Perrin D. (2014). Triggers of bulimia and compulsion attacks: validation of the “Start” questionnaire. *Encephale* 40 323–329. 10.1016/j.encep.2013.06.008 24091068

[B16] Scollon C. Kim-Prieto C. Diener E. (2003). Experience sampling: promises and pitfalls, strengths and weaknesses. *J. Happiness Stud.* 4 5–34. 10.1023/A:1023605205115

[B17] Wallis D. J. Hetherington M. M. (2004). Stress and eating: the effects of ego-threat and cognitive demand on food intake in restrained and emotional eaters. *Appetite* 43 39–46. 10.1016/j.appet.2004.02.001 15262016

[B18] White M. A. Grilo C. M. (2005). Psychometric properties of the Food Craving Inventory among obese patients with binge eating disorder. *Eat. Behav.* 6 239–245. 10.1016/j.eatbeh.2005.01.001 15854870

[B19] Witt A. A. Lowe M. R. (2014). Hedonic hunger and binge eating among women with eating disorders. *Int. J. Eat. Disord.* 47 273–280. 10.1002/eat.22171 24014479

[B20] Yeomans M. R. Blundell J. A. Leshem M. (2004). Palatability: response to nutritional need or need-free stimulation of appetite? *Brit. J. Nutr*. 92 S3–S14. 10.1079/bjn20041134 15384315

[B21] Zizza C. Siega-Riz A. M. Popkin B. M. (2001). Significant increase in young adults’ snacking between 1977-1978 and 1994-1996 represents a cause for concern! *Prev. Med.* 32 303–310. 10.1006/pmed.2000.0817 11304090

